# Bile Duct Lithiasis Mimicking a Perihilar Cholangiocarcinoma—An Endless Dilemma: A Case Report

**DOI:** 10.3390/jcm12155104

**Published:** 2023-08-03

**Authors:** Marco Piccino, Ilda Hoxhaj, Ugo Grossi, Maurizio Romano, Marco Brizzolari, Michele Scopelliti, Michele Finotti, Giacomo Zanus

**Affiliations:** 1 2nd Surgery Unit, “Ca’ Foncello” Regional Hospital, 31100 Treviso, Italy; ilda.hoxha31@gmail.com (I.H.); ugo.grossi@aulss2.veneto.it (U.G.); maurizio.romano@aulss2.veneto.it (M.R.); marco.brizzolari@aulss2.veneto.it (M.B.); michele.scopelliti@aulss2.veneto.it (M.S.); michele.finotti@aulss2.veneto.it (M.F.); giacomo.zanus@aulss2.veneto.it (G.Z.); 2 Surgical-Oncological-Gastroenterological Science Department (DISCOG), University of Padua, 35122 Padova, Italy; 3 Simmons Transplant Institute, Baylor University Medical Center, Dallas, TX 75246, USA

**Keywords:** case report, cholangiocarcinoma, intrahepatic lithiasis

## Abstract

Hilar bile duct strictures are mostly caused by malignant lesions. The morphological appearance of perihilar cholangiocarcinomas in various imaging modalities have other malignant and even benign mimics, which pose challenges to an accurate diagnosis and treatment and drive to futile surgery. Herein, we present the case of a 50-year-old woman admitted with jaundice and abdominal pain, elevated bilirubin level, liver function tests and carbohydrate antigen 19-9 level. Magnetic resonance cholangio-pancreatography (MR-CP) and the computed tomography with contrast enhancement revealed a suspected extrahepatic cholangiocarcinoma of the common bile duct. Further spontaneous resolution of the scenario, confirmed by diagnostic assessment, changed the clinical hypothesis in favor of a non-oncological disease. Indeed, the multidisciplinary evaluation supported a diagnosis of transient cholangitis secondary to non-evident intrahepatic lithiasis rather than cholangiocarcinoma. After a 26-month follow-up, the patient was asymptomatic with normal tumor markers and laboratory data. Consecutive MR-CPs showed no suspicion of malignancy. This case report underlines the need for an accurate preoperative assessment in patients with suspected cholangiocarcinoma.

## 1. Introduction

Hilar bile duct strictures are mostly caused by malignant lesions [1]. A bile duct resection with en-bloc hepatic resection is still the only curative treatment option for patients with suspected carcinoma at the hepatic confluence. Nevertheless, the 90-day postoperative mortality rate after this operation ranges from 2% to 15% in referral centers [2,3]. According to the NCCN guidelines for cholangiocarcinoma, an extensive assessment with multiple imaging techniques (ultrasonography [US], contrast-enhanced computed tomography [CT] or magnetic resonance cholangiography [MR-CP]) helps to differentiate between malignant and benign proximal bile duct strictures [4,5]. However, several authors have reported a benign stricture on the final histopathological examination of surgical specimens in 14–24% of patients after a resection for suspected perihilar cholangiocarcinoma (PHCC) [6]. A variety of benign lesions at the liver hilum may mimic malignancy, e.g., primary sclerosing cholangitis, sclerosing cholangitis IgG4 positive-lymphoplasmacytic sclerosing cholangitis, recurrent pyogenic cholangitis, inflammatory pseudotumor, impacted stones with periductal fibrosis, Mirizzi’s syndrome, or portal biliopathy [7]. Therefore, we herein describe the case of an intrahepatic lithiasis with migrating stones mimicking a PHCC. The case report has been reported in line with the SCARE criteria [8].

## 2. Case Description

A 50-year-old woman was admitted to the emergency department of a community hospital complaining of severe pain in the upper part of the abdomen and jaundice in the last few days.

As for the past medical history, the patient suffered from Hashimoto’s thyroiditis, treated with levothyroxine, and had undergone laparoscopic cholecystectomy six months earlier for gallstones disease with an uneventful postoperative course; at that time, the preoperative ultrasound of the abdomen described gallstones disease with no suspicion of bile duct lithiasis. An MRI was not performed, but preoperative and postoperative blood tests (included bilirubin, gamma-GT, alkaline phosphatase, liver enzyme levels) were reported within normal limits.

The patient (body mass index, 29.4 kg/m^2^) had no allergies and no noteworthy family history of liver or oncological diseases.

At the time of admission, blood tests were significant for total bilirubin, with 7 mg/dL, and conjugated bilirubin, with 5.5 mg/dL. The blood tests revealed increased liver enzyme levels (glutamic pyruvic transaminase (S-GPT) 325 U/L and glutamic oxaloacetic transaminase (S-GOT), 145 U/L), and a prothrombin time as international normalized ratio (PT-INR) of 1.36. The full blood count, glucose level, and electrolyte panel were within the normal range. The patient was hospitalized to further investigate the causes of jaundice. As for the serum tumor markers, the carbohydrate antigen (CA) 19-9 level was 281.5 U/mL, whereas alpha fetoprotein, carcinoembryonic antigen, and CA-125 were negative. Serological tests for chronic hepatitis B and C virus were negative.

**Figure 1:**
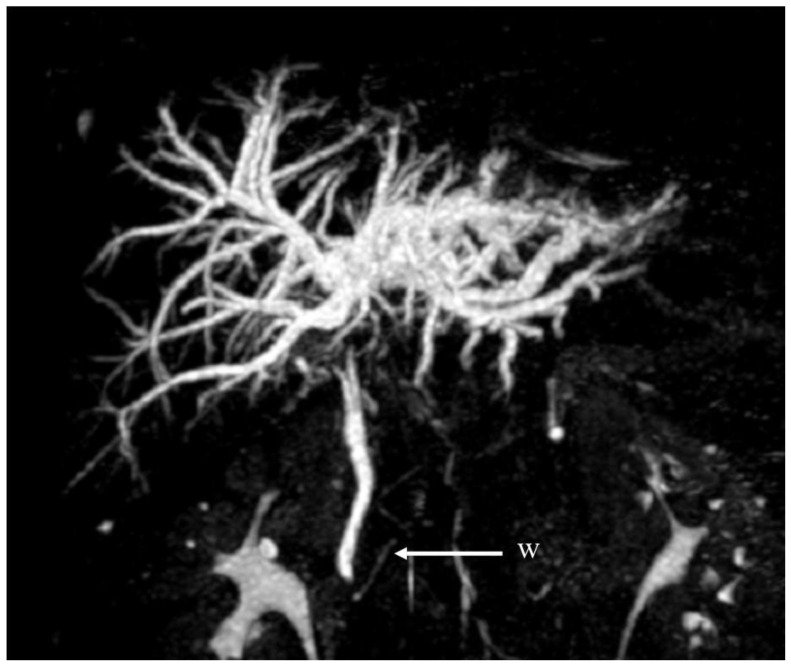
MRCP showing a stricture at the common bile duct. W, Wirsung duct.

MR-CP and MR, with contrast enhancement, described a normal choledochus with no sign of intraluminal lithiasis or a trifurcation of bile ducts, yet a relatively uncommon anatomical variant accounted for 9.3% in a study population [9]; this was displayed as a sharp stenosis with a dilation of intrahepatic bile ducts two centimeters caudally to the biliary confluence, which was more pronounced in the left hemiliver, and with no evidence of lithiasis. In a segmental biliary branch of the left hemiliver, two intraluminal defects were present. Following the MRI findings, extrahepatic cholangiocarcinoma was suspected (Figure 1).

Four days later, a chest and abdomen CT with contrast enhancement confirmed the dilation of intrahepatic bile ducts, with parietal enhancing in the portal and late phases, hepatic bile ducts at the confluence, and partial thrombosis of the left portal vein with an arterial signal enhancement of the parenchyma in the left hemiliver and in segment 8. A millimetric hypervascular area at the preampullar choledochus was also described. Some lymphadenopathies in the hepatoduodenal ligament were present, with a maximum long axis of 2.5 cm, and with short axes below 1 cm. No free fluid was described. Given the radiological imaging and oncological markers, a Klatskin type 1 tumor was suspected, with no intrahepatic or clear lymph node metastases, no local infiltration, and no macrovascular invasion. Therefore, she was immediately referred to our unit for a further preoperative assessment. On admission, the physical examination showed conjunctival jaundice without abdominal tenderness, splenomegaly, or hepatomegaly. Our multidisciplinary team, comprising even a radiologist specialized in HPB imaging, reviewed the CT and MRI performed, confirming the suspected malignancy.

Less than three days after the CT, the patient complained of an acute severe abdominal pain at the upper left quadrant radiated to the back, and had been vomiting and feeling nauseous. The vital signs, abdominal, chest, cardiovascular and neurological examinations were unremarkable. The laboratory data showed a white blood cell count of 12.7 × 10^3^/microL, INR 1.51, total bilirubin of 7.5 mg/dL, direct bilirubin of 6.9 mg/dL, S-GOT 123 U/L, S-GPT 248 U/L, amylase 1462 U/L, lipase 3215 U/L, and CA19-9 35 U/L. The scenario was suggestive for acute mild pancreatitis. The serum IgG4 sample resulted in 51 mg/dL (within normal range). Two days later, she showed persistently elevated white blood cell counts and C-reactive proteins with a fever: the nasopharyngeal swab for SARS-CoV-2 was negative, as well as blood and urine cultures. Treatment with broad spectrum antibiotics (i.v. piperacillin-tazobactam 4.5 gr TID) was initiated with subsequent clinical improvement, thus biliary drainage was not attempted. Doppler ultrasonography of the lower extremities showed no evidence of bilateral, distal or femoro-popliteal deep venous thrombosis.

**Figure 2:**
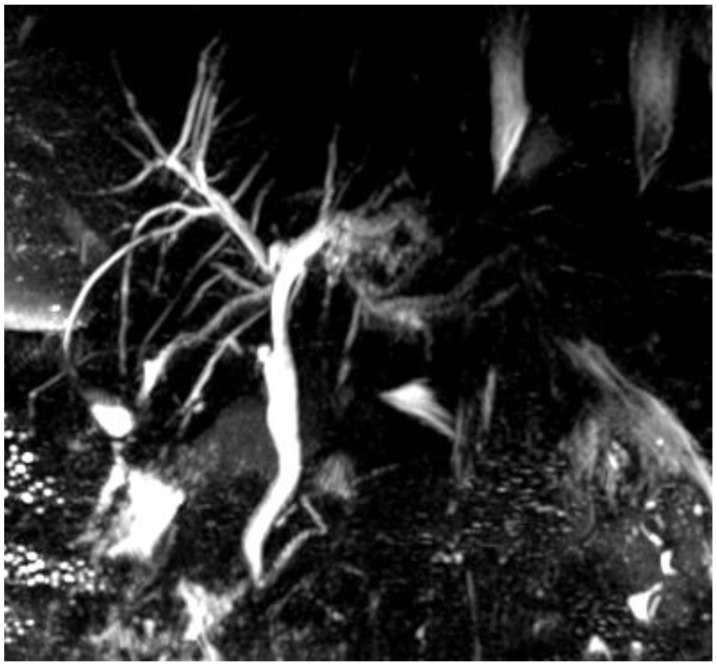
MRCP showing improvement in the intrahepatic bile duct dilatation.

The subsequent CT showed dilated intrahepatic bile ducts in the left hemiliver and S8, parietal thickening of bile ducts at the hilum and above the confluence with early arterial phase enhancing, partial portal vein thrombosis in subsegmental branches for S4 and S8, and pancreatic and peripancreatic effusion with abdominal free fluid. The MRCP and MR control two days later (Figure 2) revealed an inhomogeneous and early enhancement in the parenchymal signal in S4 and S8, likely secondary to subsegmental portal vein thrombosis; a reduction in intrahepatic biliary duct dilation, as compared to the previous MRI; and no intraluminal filling defects of the intrahepatic and extrahepatic bile ducts. The thickening of the bile duct walls had distinctly improved. No anticoagulant therapy was started yet. The intrahepatic portal vein flow restoration was assessed over the following days with Doppler ultrasonography. As a result, the non-operative management was a drive for the spontaneous improvement in the general conditions, with thrombosis resolution and a decrease in the total bilirubin and CA19-9 levels to within the normal range (Table 1).

The clinical case was presented to our multidisciplinary decision team where the suspicion of a Klatskin tumor was eventually discarded in favor of a cholangitis secondary to intrahepatic lithiasis. Probably the stones, not described at imaging, firstly stopped caudally to the biliary confluence, and secondly impacted in the distal choledochus, giving an acute pancreatitis. The patient was discharged in an optimal general condition and sent to a clinical, biochemical, and radiological follow-up. At the two-month follow-up, the MR showed intrahepatic lithiasis within S1 with regular caliber and walls of the remaining biliary tree, hypodense areas at S8 likely from the previous episodes of cholangitis, and neither suspicious focal lesion nor Wirsung dilation. The tumor marker levels had an AFP of 5.3 ng/mL, CEA of 1.0 ng/mL and CA 19-9 of 4.4 U/mL.

Moreover, during the follow-up, the patient underwent hematological tests to assess congenital thrombophilia, which resulted in negative for factor I and factor V Leiden mutations, lupus anticoagulant and anticardiolipin antibodies. However, a heterozygous MTHFR mutation was detected with normal homocysteine levels.

The multidisciplinary decision team eventually recommended conservative treatment with ursodeoxycholic acid 300 mg TID and a follow-up with an abdominal MRI every 4 months for the next two years.

After 26 months, the patient was asymptomatic, the MRs showed no suspicion of malignancy, and stable intrahepatic millimetric stones impacted in two sub-segmental ducts for S1.

## 3. Discussion

In the literature, there is limited evidence on lesions mimicking PHCC (Table 1), mostly deriving from case reports or small case series [10,11]. More precise data could be retrieved from the results of histopathological specimens in studies on the surgical treatment of PHCC if the variable matters to the outcomes, otherwise it could be hard to estimate the worldwide burden of these false positive cases. In their case series of 250 consecutive hepatic resections for suspected malignancy, Clayton et al. reported a benign histopathological entity rate in 18 (7.2%) patients [12]. The false positive rates were 2.5% among resections for colorectal metastases, 8.2% among other solid hepatobiliary tumors and 24.4% among resections for PHCC. This highlighted the difficulties in the preoperative confirmation of malignancy despite the appropriate evaluation, in particular for the hilar strictures rather than parenchymal masses. Similarly, a final pathological benign rate among patients operated on for presumed PHCC was reported by Corvera et al. (2005) [11] and Erdogan et al. [6], respectively, with 8% and 15%, thus emphasizing an emerged need for a more specific preoperative assessment.

The current standard of care [4] for the characterization of biliary strictures seen on imaging (multiphasic abdominal CT/MR with contrast enhancement, MRCP), alongside tumor markers (CEA, CA19-9), begins with endoscopic retrograde cholangiopancreatography, which utilizes fluoroscopy to create two dimensional images of the biliary system. Diagnostic options for biliary strictures or lesions include cytology brushing and intraductal biopsy [13]. Although still widely used and inexpensive, ductal cytology brushing is burdened by poor sensitivity and often yields inadequate specimens [14]. Brush cytology in the case of biliary stenosis is possible during ERCP or PTC. However, the reported sensitivity rate is around 50%, specificity 98.5%, and accuracy is near 70% [15] since the chronic inflammatory changes (cytological atypia, fibrosis, etc.) after longstanding biliary stenting may result in false positive diagnoses. Pang et al. concluded that a serum CA19-9 level > 233.15 U/mL and a CEA level > 2.98 ng/mL are more likely associated with malignant disease [16]. Surgery may be performed when the index of suspicion is high, without the need for a biopsy (NCCN, 2022). Future options for a third-level preoperative assessment, that might be deeply further developed, include the intraductal ultrasound and cholangioscopy, which have been used for an accurate description of the parietal alterations and to guide targeted biopsies. The usage of these technical assessments, although promising for their accuracy, are highly expert-dependent and still not widely available [17].

On the other side, a false negative case report described the case as well, where a PHCC was diagnosed in a patient admitted for hepatolithiasis. In that case, the biopsy of the left hepatic duct eventually suggested the presence of cholangiocarcinoma; thus, surgery was performed, and the patient was treated with a resection. The pathological examination of the specimen revealed PHCC with intra-tumoral fixed calcifications, while no stones were found [18]. Until a definitive preoperative test is available to accurately distinguish benign strictures from PHCC, a resection remains to be the treatment approach for patients with suspicious PHCC [11]. We briefly reviewed (Table 2) the peculiar aspects of benign lesions which could mimic a perihilar cholangiocarcinoma [19,20,21,22,23,24,25,26,27,28,29]; however, because of the low specificity of such aspects and a certain overlap in their presentation, further third-level assessments are needed.

In this context, the limited evidence, particularly affecting small sample size studies, adds little to the accuracy of differential diagnoses between benign and malignant biliary strictures. Our case report might help the clinicians in the decision-making process of similar cases and warn on the possibility of false positive Klatskin tumors on imaging.

## 4. Conclusions

Several non-malignant abnormalities may mimic malignant diseases of the biliary ducts, posing challenges to the differential diagnosis. We presented a case where the results of a standard preoperative assessment workup initially supported a misdiagnosis of PHCC in a patient affected by intra-/extrahepatic lithiasis. The still highly reported false positive rate in surgical specimens should force the HPB community to improve the specificity of the preoperative assessments.

## Figures and Tables

**Table 1:** Timeline of the patient’s non-operative management.

Lab Tests							
Total bilirubin (mg/dL)	7			7.5	3.2	2	1.2
Direct bilirubin (mg/dL)	5.5			6.9	2.7	1.8	1.2
S-GPT (U/L)	325			248	80	55	49
S-GOT (U/L)	145			123	24	31	30
ALP (U/L)	324			387	248	224	161
Lipase (U/L))	41			3215	82	77	37
PT-INR	1.36			1.51	1.32	1.18	1.13
Hb (g/dL)	13.5			14.3	11.9	12.3	12.7
Leucocytes (cell/mcl)	10,510			12,710	12,640	13,540	5070
CRP (mg/dL)	0.96			2.8	26.2	17.96	0.52
CA19-9 (U/mL)	281.5			35			8.6
Imaging performed	Abdominal MRI + MRCP	Abdominal CT			Abdominal CT	Abdominal MRI + MRCP	
Events	Admission to community hospital		Admission to our Dept.	Acute pancreatitis			Resolution of the scenario
Timeline	Day 0	4 days later	6 days	7 days	10 days	12 days	21 days

*Abbreviations:* MRCP, magnetic resonance cholangiopancreatography; MRI, magnetic resonance imaging; CT scan, computed tomography scan; ALP, alkaline phosphatase; GOT, glutamic oxaloacetic transaminase; GPT, glutamic pyruvic transaminase; PT-INR, prothrombin time International Normalized Ratio; Hb, Hemoglobin; CRP, c-reactive protein; CA19-9, carbohydrate antigen 19-9.

**Table 2:** Benign lesions mimicking a perihilar cholangiocarcinoma: clinical differential diagnostic criteria.

	Primary Sclerosing Cholangitis	Lymphoplasmacytic Sclerosing Cholangitis-Sclerosing Cholangitis IgG4 Positive (as Part of Systemic IgG4-Related Disease)	Recurrent Pyogenic Cholangitis	Inflammatory Pseudotumor (IPT)	Impacted Stones with Periductal Fibrosis	Mirizzi Syndrome	Portal Biliopathy
**Clinincal presentation**	Jaundice, pruritus, abdominal pain.	Jaundice, pruritus, abdominal pain.	Jaundice, fever, abdominal pain.	Asympthomatic or abdominal pain and fever.	Jaundice, fever, abdominal pain.	Jaundice, ± fever, abdominal pain.	Often asympthomatic. Patient history of extrahepatic portal vein thrombosis is the most common cause of portal biliopathy; rare in cirrhosis, portal vein fibrosis without cirrhosis and congenital hepatic fibrosis.
**Epidemiology**	Age at diagnosis 44.2 y.o. ± 17.4 (11–81) *. The age-adjusted incidence rate for males was numerically greater than females. Patients are usually nonsmokers, and about 2/3 have a coexistent IBD (75% ulcerative colitis).	Middle to upper age, with an onset at 50–70 years, Male:female ratio = 3:7.	More frequent between the third and fifth decades.			Incidence correlates to gallbladder stones.	
**Laboratory test**	Elevated ALP, GGT, bilirubin. *Different non-specific autoantibodies* correlate with PSC, such as P-ANCA, ANA, anti-smooth muscle autoantibodies	Elevated serum IgG4 concentration (≥135 mg/dL). CA19-9 can be elevated.		Elevated inflammatory markers (including erythrocyte sedimentation rate, C-reactive protein, and leukocyte count) are common. CA19-9 usually normal.		Elevated ALP, GGT, bilirubin. ± elevated inflammatory markers. CA19-9 can be elevated because of jaundice.	Elevated ALP, GGT, bilirubin, GOT, GPT. CA19-9 can be elevated.
**Appropriate** **imaging modality and features**	MRCP, ERC. Beaded appearance, pruned tree appearance, and band-like stricture.	MRCP. Diffuse or segmental narrowing of the intrahepatic and/or extrahepatic bile duct, associated with the thickening of the bile duct wall.	MRCP. Intraductal calculi and bile duct strictures.	CT-scan, MRI. The CT-scan: lesions with variable c.e., may present as hypovascular with delayed enhancement because of fibrosis. The MRI may produce hypointense on T1 sequences with moderate-to-high hyperintense on T2 sequences.	CT scan (scarce sensitivity for non-calcific stones) and MRCP.	CT scan, MRCP. MRCP most accurate, shows an extrinsic narrowing of the common hepatic duct, a gallstone in the cystic duct, dilation of the intrahepatic and common hepatic ducts, with a normal common bile duct.	CT scan and portal MR and MRCP. Show portal cavernoma, paracholedochal and/or epicholedochal dilations, portosystemic shunts and abnormal morphology of the bile duct.
**Specific investigation**	Cholangiography, liver biopsy for doubtful cases.	Elevated serum IgG4 concentration (≥135 mg/dL).		Exclusion diagnosis. Percutaneous liver biopsy.			
**Histo-pathological examination**	Obliterative, non-suppurative cholangitis with substantial periductular fibrosis, referred to as “onion-skin fibrosis”.	Marked lymphocytic and plasmacytic infiltration and fibrosis. Infiltration of IgG4-positive plasma cells (>10 cells per high-power field). Storiform fibrosis. Obliterative phlebitis.	Chronic, recurrent infections from parasites predispose to the development of pigmented calculi, cholangitic abscesses, and inflammatory strictures.	Inflammatory infiltrate consisting of lymphocytes, plasma cells, and histiocytes admixed with a variable proportion of fibroblasts and myofibroblasts.			Acute or chronic cholecystitis.
**Specific medical intervention**	High dose of UDCA (20 mg/kg/d) improve liver function tests.	Prednisone 20–40 mg/d.		Improvement after antibiotics or after steroid administration.			Treat underlying disease.

* Expressed as mean ± standard deviation (range). *Abbreviations:* ERC, endoscopic retrograde cholangiography; MRCP, magnetic resonance cholangiopancreatography; MRI, magnetic resonance imaging; CT scan, computed tomography scan; IBD, Inflammatory Bowel Disease; ALP, alkaline phosphatase; GGT, gamma glutamyltransferase; GOT, glutamic oxaloacetic transaminase; GPT, glutamic pyruvic transaminase; c.e., contrast enhancement; UDCA, ursodeoxycholic acid; P-ANCA, perinuclear antineutrophil cytoplasmic antibodies; ANA, antinuclear antibodies.

## Data Availability

No new data were created or analyzed in this study. Data sharing is not applicable to this article.
